# Surface Tension and Adsorption Studies by Drop Profile Analysis Tensiometry

**DOI:** 10.1007/s11743-017-2016-y

**Published:** 2017-09-04

**Authors:** T. Kairaliyeva, E. V. Aksenenko, N. Mucic, A. V. Makievski, V. B. Fainerman, Reinhard Miller

**Affiliations:** 1 Max-Planck-Institut für Kolloid-und Grenzflächenforschung, Potsdam, Germany; 2 Institute of Colloid Chemistry and Chemistry of Water, Kyiv (Kiev), Ukraine; 3 Faculty of Technology, University of Novi Sad, Novi Sad, Serbia; 4 SINTERFACE Technologies, Berlin, Germany

**Keywords:** Bubble and drop profile analysis tensiometry, Surfactant adsorption layers, Surfactant depletion due to adsorption

## Abstract

Surface tension and dilational viscoelasticity of solutions of various surfactants measured with bubble and drop profile analysis tensiometry are discussed. The study also includes experiments on the co-adsorption of surfactant molecules from a solution drop and alkane molecules from saturated alkane vapor phase. Using experimental data for 12 surfactants with different surface activities, it is shown that depletion due to adsorption of surfactant from the drop bulk can be significant. An algorithm is proposed quantitatively to take into consideration the depletion effect which is required for a correct description of the co-adsorption of alkanes on the solution drop surface and the correct analysis of experimental dynamic surface tension data to determine the adsorption mechanism. Bubble and drop profile analysis tensiometry is also the method of choice for measuring the dilational viscoelasticity of the adsorbed interfacial layer. The same elasticity moduli are obtained with the bubble and drop method only when the equilibrium surface pressures are sufficiently small (Π < 15 mN m^−1^). When the surface pressure for a surfactant solution is larger than this value, the viscoelasticity moduli determined from drop profile experiments become significantly larger than those obtained from bubble profile measurements.

## Introduction

Studies of surface (interfacial) tension and adsorption of surfactants and polymers (proteins) at liquid/fluid interfaces constitute an important branch of surface science. They aim, for example, to find relationships between the properties at interfaces and the structure of adsorbed surfactants [1–6]. Also, the design of optimum formulations containing mixtures of surfactants is a challenging target [7–9].

Various methods used in these studies, which have been extensively reviewed in, make it possible to determine the equations of state of surface layers and corresponding adsorption isotherms of surfactant solutions [10, 11]. These models express the surface tension and adsorption of components as functions of the surface layer composition. Drop and bubble profile analysis tensiometry have been widely used by various authors into study surface tension and dilation viscoelasticity of various systems, such as aqueous solutions of surfactants, proteins, or their mixtures [12–36]. These methods provide relatively simple procedures to obtain important experimental data which characterise the surface (adsorption) activity of substances, the competitive adsorption and interrelation between surfactants and various additives in mixed solutions. The volume of studied solutions could be as low as a few millilitres, which is important in medical applications intended to study various biologic liquids (such as blood serum, amniotic fluid, gastric juice, saliva, endocrine glands, cerebrospinal, and alveolar lining fluid) available often only in small amounts [37, 38].

The applicability of the drop profile method, if used for the quantitative analysis of adsorption models (equations of state of adsorption layer and adsorption isotherm), is confined to surfactants which exhibit relatively weak surface activity. This is because in solutions of highly surface-active surfactants the concentration within the drop bulk becomes essentially lower as compared to the initial concentration due to the adsorption of the surfactant molecules at the drop surface. Therefore, if this effect is neglected, the analysis of the surface tension and adsorption isotherms as functions of the initial surfactant concentration becomes erroneous. On the other hand, this feature of the drop profile analysis method could be an advantage if the method is used in combination with the bubble profile (or du Noüy ring, or Wilhelmy plate method), where the volume of the studied solution is large, and therefore, any surfactant depletion due to adsorption does not occur. By a comparative processing of the results from drop and bubble profile analysis tensiometry, the adsorbed amount can be directly determined [39–43]. In particular, it has been shown that this procedure represents a new method for the direct determination of the adsorbed amounts for highly surface active surfactants and proteins. The results of dilation rheological studies using the bubble and drop profile tensiometry have been compared [44–48]. Of special interest are the results obtained in studies of the adsorption of alkanes from the gaseous phase on the drop of water and/or aqueous solutions of proteins and surfactants as reported in [49–53].

In this review, we present a theoretical protocol together with experimental data from the literature to demonstrate that it is possible to determine the parameters of model equations of state and adsorption isotherms for various surfactant solutions using drop profile analysis tensiometry, to verify the diffusional adsorption model for the drop method, the model of co-adsorption of alkane from gaseous phase and surfactant (protein) from the drop bulk, and the model which describes the dilation rheology for drop and bubble surface layers. To verify the proposed approach, experimental results for 12 different surfactants were analysed. In the above sequence, the adsorption activity of the surfactants increases, and therefore the differences between the results obtained by bubble and drop profile analysis tensiometry become increasingly evident.

## Experimental

### Materials and Methods

In this study, the earlier published results obtained for solutions of various surfactants are mainly used; in some cases additional experiments were also performed. The non-ionic surfactants discussed in this study are widely used for both scientific and industrial purposes. Decanol (C_10_OH); alkyl dimethyl phosphine oxides (C_13_DMPO and C_11_DMPO); polyoxyethylene (20) sorbitan monolaurate (Tween™ 20, C_26_H_50_O_10_); polyethylene glycol octylphenyl ethers (Triton™ X-45 and Triton™ X-100, C_14_H_21_O(C_2_H_4_O)_n_H); and the ethoxylated alcohols C_10_EO_8_, C_12_EO_5_ and C_14_EO_8_ were purchased from Sigma Chemical and used without further purification.

The ionic surfactants used in the study are CTAB (hexadecyl trimethyl ammonium bromide) and SDS (sodium dodecyl sulphate). The CTAB solutions were studied in phosphate buffer solutions (0.01 M, pH 7, prepared by mixing appropriate stock solutions of Na_2_HPO_4_ and NaH_2_PO_4_). CTAB solutions mixed with proteins were also prepared with this buffer. The results obtained for the phospholipid 1-*O*-octadecyl-2-*O*-(2-(myo-inositolyl)-ethyl)-sn-glycero-3-(R/S)-phosphatidylcholine, abbreviated by Inositol-C2-PAF are also discussed.All measurements were performed with bubble/drop profile analysis tensiometers (PAT-1 and PAT-2P, SINTERFACE Technologies, Germany) [39, 40].

### Theory

#### Adsorption Equilibrium and Kinetics Assuming a Bulk/Surface Mass Balance

We begin with the analysis of experimental data obtained using the drop profile analysis method assuming the mass balance in equilibrium conditions, referring to the results reported previously [54]. In this overview, we mainly apply the Frumkin model, which was generalised to account also for the intrinsic compressibility of the adsorbed surfactant layer. It should be noted that the Frumkin model is capable of a sound description of various adsorption systems, in particular, solutions of non-ionic surfactants. This model is also applicable to solutions of ionic surfactants with added electrolytes. For the solutions of non-ionic surfactants with long ethylene oxide chain the reorientation model is more suitable; for this case, we propose a version of the model which accounts for the mass balance in a drop of the surfactant solution with reorientable molecules in the surface layer.

The surface layer equations of state and adsorption isotherm for the Frumkin model are as follows:

1 $$- \frac{{\varPi \omega_{0} }}{RT} = \ln \left( {1 - \theta } \right) + a\theta^{2}$$

2 $$bc = \frac{\theta }{1 - \theta }\exp \left( { - 2a\theta } \right)$$

3 $$\omega = \omega_{0} \left( {1 - \varepsilon \varPi \theta } \right),$$

where Π = *γ*_0_–*γ* is the surface pressure, *γ*_0_ is the surface tensions of the solvent (water) and *γ* is the surface tensions of the studied solution, *θ* is the surface coverage (*θ* = Γ*ω*), *R* is the gas law constant, *T* is the absolute temperature, *b* is the adsorption activity coefficient, *a* is the intermolecular interaction coefficient, Γ is the adsorption, *ω*_0_ is the surfactant molar area, *ε* is the intrinsic compressibility coefficient (which for most surfactants is within the range of 0.004–0.01 m mN^−1^) and *c* is the subsurface surfactant concentration. Note, the interaction-related coefficient *a* in the Frumkin model can also be adjusted to reflect other factors: for example, the reorientation of molecules with a large number of ethylene oxide groups in the surface layer can be described by negative values of this coefficient. In the simplest case, the subsurface concentration after equilibration is assumed to be equal to the initial concentration of the adsorbed surfactant *c*_0_ in the solution bulk, and the approximate adsorption isotherm equation reads:

2a $$bc_{0} = \frac{\theta }{1 - \theta }\exp \left( { - 2a\theta } \right).$$

However, some amount of the surfactant is adsorbed at the interface, which results in a depletion of the surfactant solution. This effect is especially significant for the adsorption of surfactants from the bulk of the drop onto its surface. To account for this depletion, one should note that the mass *M* of the surfactant in the volume *V* of the initial solution with concentration *c*_0_ is equal to the sum of its amount in the volume *V* and the amount adsorbed at the interface of area *S* after equilibration: $M = c_{0} V = cV + \Gamma S$. Thus, one has:

4 $$c = c_{0} - \left( {{S \mathord{\left/ {\vphantom {S V}} \right. \kern-0pt} V}} \right)\Gamma .$$

and Eq. (2) turns into:

5 $$b\left( {c_{0} - \frac{S}{V}\Gamma } \right) = \frac{\theta }{1 - \theta }\exp \left( { - 2a\theta } \right).$$

Note that in Eq. (4), *c* is the surfactant concentration in the solution bulk within the drop, which becomes equal to the subsurface concentration only when the adsorption equilibrium is established. The solution of the set of Eqs. (1), (3), and (5) should yield the dependencies of Π and Γ on *c*_0_, which are measured in the experiment, and varying the model parameters one can try to obtain the best fit between the calculated values and experimental data, as explained previously e.g. [55, 56].

The procedure to determine the adsorption directly is as follows. When in two experiments (drop and bubble) the surface tensions *γ* are equal to each other, the adsorption values Γ are also equal. Then the Γ value can be calculated from the difference between the initial concentration within the drop *c*_D_ and the concentration in the solution around the bubble *c*_B_:

6 $$\Gamma \left( {\frac{{S_{\text{D}} }}{{V_{\text{D}} }} - \frac{{S_{\text{B}} }}{{V_{\text{B}} }}} \right) = (c_{\text{D}} - c_{\text{B}} )_{{\gamma = {\text{const}}}} ,$$

where *S*_D_ is the surface area of the drop, *V*_D_ is the solution volume inside the drop, *S*_B_ is the area of the bubble, and *V*_B_ is the volume of the solution surrounding the bubble. Typically, *V*_D_/*S*_D_ = 0.5–0.7 mm, while for the bubble profile method this ratio is very large, *V*_B_/*S*_B_ > 500 mm. Therefore, the second term in the parentheses in the left hand side of Eq. (6), can usually be neglected to obtain [39–43]:

7 $$\Gamma = \frac{{V_{\text{D}} }}{{S_{\text{D}} }}\left( {c_{\text{D}} - c_{\text{B}} } \right)_{{\gamma = {\text{const}}}} .$$

Instead of the bubble profile method, the ring or plate tensiometry can be used, because for these methods the *V*/*S* ratio is usually also large, i.e. about 20–50 mm.

It should be noted that, two papers [39] and [57] were published almost simultaneously in which the surfactant mass balance within the contacting liquid phases was studied assuming adsorption-related losses. Various experimental methods used for surface tension measurements (drop profile, du Noüy ring and Wilhelmy plate) were analysed and equations similar to Eq. (7) were derived.

Various adsorption mechanisms have been discussed in the literature. To calculate the dynamic surface tensions, the diffusion controlled adsorption model based on Fick’s equation was employed [58]. The diffusion of the surfactant in a drop of radius *R* is governed by Fick’s law. In spherical coordinates it reads:

8 $$\frac{\partial c}{\partial t} = D\left( {\frac{{\partial^{2} c}}{{\partial r^{2} }} + \frac{2}{r}\frac{\partial c}{\partial r}} \right)\quad {\text{for }}0 < r < R,$$

where *c* = *c*(*r*, *t*) is the surfactant concentration as a function of time *t* and distance *r* measured from the drop centre, and *D* is the surfactant’s diffusion coefficient in the drop phase. The adsorption Γ as a function of time is given by the diffusion flux:

9 $$\frac{{{\text{d}}\Gamma }}{{{\text{d}}t}} = - \left. {D\frac{\partial c}{\partial r}} \right|_{{r = R^{ - } }} .$$

Equation (9) serves as boundary condition at the interface, which is located at *r* = *R*. Note, this is the moment when the adsorption isotherm enters the calculations, because it determines the dependence between Γ and *c*. A second necessary boundary condition, defined in the drop centre at *r* = 0, results from the symmetry of the spherical drop and the fact that the diffusion happens in a closed system:

10 $$\left. {\frac{\partial c}{\partial r}} \right|_{r = 0} = 0$$

For the experiments used in this study, we can assume a homogeneous initial distribution of the surfactant. Hence, the initial condition for Eq. (9) is:

11 $$c\left( {r,0} \right) = c_{\text{D}} \quad {\text{for }}0 < r < R.$$

Software was developed to calculate the dependencies of surface tension, adsorption, surface coverage, subsurface concentration, and average concentration in the drop bulk on time. At long adsorption times, the average concentration is the equilibrium concentration which is equal to the bulk concentration of the ambient solution in the bubble profile method, provided the equilibrium surface tension at the surface of the bubble and the drop are identical.

In our experiments the drop volume *V* for different surfactants was in the range of 24–26 mm^3^, while the drop surface area *S* was 35–38 mm^2^; therefore, the ratio *S*/*V* was 1.42–1.58 mm^−1^. For a sphere we have *S*/*V* = 3/*r*, thus the radius of a corresponding spherical drop is *R* = 1.9–2.0 mm; this value was assumed in the adsorption kinetics calculations using Fick’s Eq. (8).

#### Reorientation of Adsorbed Molecules

A good description of the adsorption behaviour of various surfactants can be obtained by the Frumkin model. For ethoxylated surfactants, however, especially those with large ethoxylated head groups and hydrocarbon chains, the Frumkin model oversimplifies the adsorption behaviour and the so-called reorientation model provides a better description of the interfacial layer. The main assumption in this model is that the adsorbed surfactants can have two orientations (subscripts 1 and 2) [58]. The resulting equation of state reads:

12 $$- \frac{{\varPi \omega_{0} }}{RT} = \ln (1 - \Gamma \omega ) + \Gamma (\omega - \omega_{0} ) + a(\Gamma \omega )^{2} ,$$

where $\omega = {{\left( {\omega_{1} \Gamma_{1} + \omega_{2} \Gamma_{2} } \right)} \mathord{\left/ {\vphantom {{\left( {\omega_{1} \Gamma_{1} + \omega_{2} \Gamma_{2} } \right)} \Gamma }} \right. \kern-0pt} \Gamma }$ is the average molar area with $\theta = \omega \Gamma = \omega_{1} \Gamma_{1} + \omega_{2} \Gamma_{2}$ being the surface coverage, and $\Gamma = \Gamma_{1} + \Gamma_{2}$ being the total adsorption. The two molar areas *ω*_1_ and *ω*_2_ refer to two orientations of adsorbed molecules. When we assume that *ω*_2_ > *ω*_1_ and *ω*_1_ = *ω*_0_(1 − *ε*Π*θ*), with *ω*_0_ being the molar area of the surfactant at zero surface coverage (or alternatively, *ω*_0_ being the molar area of the solvent), we obtain the adsorption isotherms for the adsorption states:

13 $$bc = \frac{{\Gamma_{1} \omega_{0} }}{{\left( {1 - \Gamma \omega } \right)^{{\omega_{1} /\omega_{0} }} }}\exp \left( { - 2a\Gamma \omega \frac{{\omega_{1} }}{{\omega_{0} }}} \right)$$

14 $$bc = \frac{{\Gamma_{2} \omega_{0} }}{{(\omega_{2} /\omega_{1} )^{\alpha } \left( {1 - \Gamma \omega } \right)^{{\omega_{2} /\omega_{0} }} }}\exp \left( { - 2a\Gamma \omega \frac{{\omega_{2} }}{{\omega_{0} }}} \right),$$

where *α* is the exponent of a power law, which accounts for different surface activities of the molecules in the two adsorption states, and similarly to the definitions in the previous section, *c* is the subsurface concentration of the surfactant, and *b* is the coefficient defining the adsorption equilibrium. The ratio of the adsorption values for the two states of the molecules in the interfacial layer is obtained from Eqs. (13) and (14):

15 $$\frac{{\Gamma_{1} }}{{\Gamma_{2} }} = \frac{{(\omega_{1} /\omega_{2} )^{\alpha } }}{{\left( {1 - \Gamma \omega } \right)^{{(\omega_{2} - \omega_{1} )/\omega_{0} }} }}\exp \left( { - 2a\Gamma \omega \frac{{(\omega_{2} - \omega_{1} )}}{{\omega_{0} }}} \right).$$

This expression is the physico-chemical principle of Braun-Le Châtelier, which was introduced by Joos to describe the behavior of adsorption layers [11].

Previously a model was proposed for surfactant molecules adsorbed at the surface in any number of states; the molar area of molecules in different states are different, and the areas of neighbouring states differ from each other by the area increment *ω*_0_ [59]. The equation of state for this model which we refer to as the n-state model is similar to Eq. (12):

16 $$- \frac{{\varPi \omega_{1} }}{RT} = \ln (1 - \Gamma \omega ) + \Gamma (\omega - \omega_{1} ) + a(\Gamma \omega )^{2} ,$$

where *ω*_1_ is the molar area corresponding to the state with minimum molar area. The adsorption isotherm for the *i*-th state is:

17 $$bc = \frac{{\Gamma_{i} \omega_{1} \exp \left[ { - \frac{{\omega_{i} }}{{\omega_{1} }}(2a\Gamma \omega )} \right]}}{{\left( {{{\omega_{i} } \mathord{\left/ {\vphantom {{\omega_{i} } {\omega_{1} }}} \right. \kern-0pt} {\omega_{1} }}} \right)^{\alpha } (1 - \Gamma \omega )^{{\omega_{i} /\omega_{1} }} }}.$$

the distribution of adsorptions over the states with different molar areas is determined by the equation:

18 $$\Gamma _{i} = \Gamma \frac{{\left( {\frac{{\omega _{i} }}{{\omega _{1} }}} \right)^{\alpha } \left( {1 - \Gamma \omega } \right)^{{\frac{{\omega _{i} - \omega _{1} }}{\omega }}} \exp \left[ {\left( {\frac{{\omega _{i} - \omega _{1} }}{\omega }} \right)\left( {2a\Gamma \omega } \right)} \right]}}{{\sum\nolimits_{{i = 1}}^{n} {\left( {\frac{{\omega _{i} }}{{\omega _{1} }}} \right)^{\alpha } \left( {1 - \Gamma \omega } \right)^{{\frac{{\omega _{i} - \omega _{1} }}{\omega }}} \exp \left[ {\left( {\frac{{\omega _{i} - \omega _{1} }}{\omega }} \right)\left( {2a\Gamma \omega } \right)} \right]} }}$$

Here *ω*_i_ = *ω*_1_ + (*i* − 1)*ω*_0_, $$n = \frac{{\omega_{m} - \omega_{1} }}{{\omega_{0} }} + 1$$, $$\Gamma = \sum\nolimits_{{i = 1}}^{n} {\Gamma _{i} }$$, $$\omega \Gamma = \sum\nolimits_{{i = 1}}^{n} {\omega _{i} \Gamma _{i} }$$.

The model defined by Eqs. (16)–(18) is quite similar to the model developed to describe the adsorption of proteins This model for the adsorption of proteins from aqueous solutions assumes *n* states for the adsorbed protein molecules having molar areas between a maximum value (*ω*_max_) at very low surface pressure Π (or low surface coverage *θ*) to a minimum value *ω*_min_ at high surface pressure/coverage. The difference between the molar areas of two “neighboring” protein conformations is again given by the molar area increment *ω*_0_.

#### Adsorption of Surfactants from Inside the Drop and from the Surrounding Ambient Phase

There are several studies on adsorption layers formed from mixed surfactant solutions. Rosen and coworkers were pioneers in this field [7, 8, 60–62]. We have further developed the model to describe mixed surface layers of a reorientable surfactant adsorbed from inside the drop, and a surfactant adsorbed from the ambient phase around the drop surface, in particular, for the adsorption of alkanes from the vapor phase surrounding the drop. For the case when the adsorption of a protein or long-chain surfactant from the solution drop occurs with the formation of the first adsorption layer, accompanied with the formation of an adjacent secondary layer formed exclusively by alkane molecules, a theoretical description was formulated [63–65]. Approximate expressions for polylayer adsorption of alkanes were proposed [53, 63, 65].

The theoretical model for the competitive adsorption process at the drop surface was defined as follows. We can assume that the transport of the surfactant inside the drop is governed by diffusion given by Eq. (8). However, if we also assume a diffusion controlled adsorption of the alkane molecules from the vapor phase we obtain very low diffusion coefficients which are 7–8 orders of magnitude too low. Thus, we proposed an alternative model in which we considered a kinetic-controlled adsorption mechanism [65]. Note, there are alternative non-diffusional mechanisms discussed in literature [66–68]. More rigorous kinetic models to describe the formation of adsorbed multi layers are presently under development.

### Dilation Rheology of Surfactant Adsorption Layers

The surface dilational viscoelasticity *E* is defined as the surface tension increase Δ*γ* for a small relative surface area increase Δ*A/A, i.e.*

19 $$E = \left. {\frac{{{\text{d}}\,\gamma }}{{{\text{d}}\,\ln A}}} \right|_{\Gamma } .$$

The viscoelastic modulus *E* can best be presented in the complex domain *E* = *E*_*r*_ + *iE*_*i*_ with *E*_*r*_ and *E*_*i*_ being the real and imaginary parts of *E*. For surfactant adsorption layers we can assume a pure diffusion controlled relaxation mechanism. Then, the real and imaginary parts of *E* are given by the relations [69, 70]:

20 $$E_{r} \left( \varpi \right) = E_{0} \frac{1 + \zeta }{{1 + 2\zeta + 2\zeta^{2} }},\quad E_{i} \left( \varpi \right) = E_{0} \frac{\zeta }{{1 + 2\zeta + 2\zeta^{2} }},$$

where $\zeta = \sqrt {\varpi_{\text{D}} /2\varpi }$, the limiting (high frequency) elasticity is $E_{0} \left( c \right) = - {\text{d}}\gamma /{\text{d}}\ln \Gamma$, and the characteristic frequency of a diffusional relaxation is defined by $\varpi_{\text{D}} \left( c \right) = D \cdot \left( {{\text{d}}c/{\text{d}}\Gamma } \right)^{2}$. The quantities *c*, *Γ*, and *D* are the surfactant bulk concentration, its adsorption and bulk diffusion coefficient, respectively. $\varpi$ is the angular frequency of the generated surface area oscillations. From Eq. (20), we can obtain suitable expressions for the viscoelasticity modulus $\left| E \right|$ and the phase angle ϕ between stress (d*γ*) and strain (d*A*):

21 $$\left| E \right| = E_{0} (1 + 2\zeta + 2\zeta^{2} )^{ - 1/2} ,\quad \phi = {\text{arctg}}\left[ {\zeta /(1 + \zeta )} \right].$$

Note, Eqs. (20) and (21) have been derived only for the relaxation of surfactants at flat surfaces. However, at low perturbation frequencies and for small spherical bubbles/drops (in classical drop and bubble experiments the radii are between 1.5 and 2 mm) the real geometry plays an important role. The corresponding equations for spherical drops/bubbles were derived Joos [11]. The adsorption from a solution at a bubble surface is given by:

22 $$E(\varpi ) = E_{0} \left\{ {1 - i\frac{D}{{\varpi r_{0} }}\frac{{{\text{d}}c}}{{{\text{d}}\Gamma }}\left( {1 + nr_{0} } \right)} \right\}^{ - 1} .$$

For the adsorption from inside a drop:

23 $$E(\varpi ) = E_{0} \left\{ {1 - i\frac{D}{{\varpi r_{0} }}\frac{{{\text{d}}c}}{{{\text{d}}\Gamma }}\left[ {nr_{0}\coth \left( {nr_{0} } \right) - 1} \right]} \right\}^{ - 1} ,$$

where $n^{2} = i\omega /D$, $\varpi$  = 2π*f*, *r*_0_ is the drop/bubble radius and *f* is the oscillation frequency.

Equations (22) and (23) are identical to Eqs. (20) for high values of *r*_0_ and $\varpi$. For low oscillation frequencies (below 0.1 Hz), however, the use of Eq. (22) leads to a decrease of the modulus |E| and an increase of the phase angle ϕ for bubbles (when compared to a flat surface), while for drops the use of Eq. (23) leads to an increase in |*E*| and a decrease in ϕ. The protocol for determining the rheological quantities was discussed in [46, 71].

## Results and Discussion

### Equilibrium Surface Tension Measured by Drop Profile Analysis Tensiometry

The figures presented in this section illustrate the dependencies of equilibrium surface tension on surfactant concentration for 12 different surfactants measured using drop and bubble profile tensiometry and analysed according to the models explained previously. It should be noted that, for the sake of comparison, the experimental points and theoretically calculated curves plotted as dependencies both on the initial bulk concentration *c*_0_, and on the equilibrium subsurface concentration *c* are shown in the same figures. To distinguish between these, the values plotted against *c*_0_ are shown in red, while those plotted against *c* are shown in black. The abscissa axis is labelled in colour to help distinguish between the values plotted in the figures vs *c*_0_ and *c*. Also, to facilitate the understanding of black/white version of this article, the data plotted vs. *c*_0_ are shown by bold lines and filled symbols, while the data plotted vs. *c* are shown by thin lines and open symbols.

Also, for the sake of brevity the values either measured by drop profile experiments (i.e. the values measured with respect to the initial bulk concentration *c*_0_) or calculated by fitting the theoretical curves to these experimental values are referred to as “drop-based values”; similarly, the values either measured by bubble profile experiments (or du Noüy ring or Wilhelmy plate experiments, which also assume that the subsurface equilibrium concentration of the surfactant *c* is equal to its concentration in the ambient volume bulk *c*_0_), i.e. the values measured with respect to the equilibrium subsurface concentration *c*, or calculated by fitting the theoretical curves to these experimental values are referred to as “bubble-based values”.

The Frumkin model parameters estimated by fitting the values calculated using Eqs. (1), (3), and (5) to the experimental data measured by the drop profile method are listed in Table 1, and ranged according to the increase of the adsorption activity coefficient *b*. Note, the model parameters estimated in [54] by fitting the values calculated using Eqs. (1)–(3) to the bubble-based experimental data are close to those summarized in Table 1. Table 1 also shows the diffusion coefficients *D* estimated by fitting the values obtained using Eqs. (8)–(11) to the dynamic surface tension dependencies. These values are shown only for the surfactants which were studied in dynamic experiments and for which the theoretical calculations were performed.

**Table 1:** Model parameters for the surfactants obtained with the Frumkin adsorption model using the drop experiments

Surfactant	*ω*_0,_ 10^5^ m^2^ mol^−1^	*b,* 10^3^ m^3^ mol^−1^	*a*	*ε*, 10^−3^ m mN^−1^	*D*, 10^−10^ m^2^ s^−1^
SDS	2.9	0.0003	1.2	5	–
SDS + NaCl	2.8	0.025	1.0	5	–
C_10_OH	2.1	0.049	1.2	3	–
C_11_DMPO	3.6	0.14	0.7	5	3.0
C_10_EO_8_	4.0	0.35	0.2	9	3.0
CTAB in buffer	3.6	0.40	0.6	9	–
Tween 20	2.5	0.83	0.6	5	–
C_13_DMPO	3.1	1.71	0.4	5	3.5
Tr-45	3.8	2.65	0.66	5	3.0
C_12_EO_5_	3.5	16.0	−0.9	5	3.8
Tr-100	3.9	32.8	−2.7	8	4.0
C_14_EO_8_	3.0	90.0	−4.7	6	2.0
Inositol	3.2	158.0	1.6	11	0.4

**Fig. 1:**
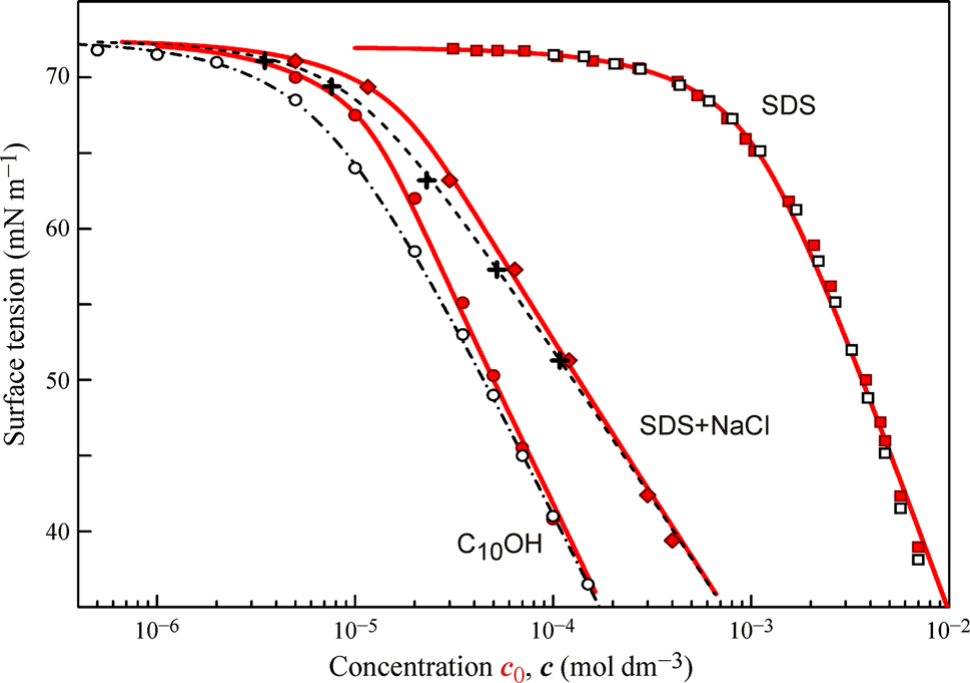
Surface tension isotherms for solutions of SDS in pure water, in water with addition of 0.5 mol dm^−3^ NaCl, and for aqueous solutions of C_10_OH; details are given in the text

The adsorption isotherm of SDS solutions in pure water measured by bubble profile tensiometry, du Noüy ring and Wilhelmy plate methods (the data reported in [72, 73]), for which the bulk concentration is close to the equilibrium subsurface one, is shown in Fig. 1 by filled squares, while the data taken from [74] and obtained with drop profile analysis tensiometry are shown by open squares. It is seen that the drop-based and bubble-based data are very similar; also, the fitting of the theoretical curves calculated using the Frumkin model for an electroneutral surface layer [73], using either Eqs. (1), (3), (5), or Eqs. (1)–(3) leads to almost coincident results because of the high concentration and low surface activity of SDS (red solid curve).

The addition of 0.5 mol dm^−3^ NaCl leads to an increased surface activity of SDS by almost two orders of magnitude (filled diamonds, drop profile data [73]); note that in the fitting calculations (shown by the bold red curve) the *ω*_0_ values listed in Table 1 refer to the total molar area of SDS. The black dashed curve was obtained by the recalculation of the fitting curve onto the equilibrium concentration; it is seen that the equilibrium surfactant concentration inside the drop is essentially lower than the initial one: e.g. at the surface tension of 70 mN m^−1^ this amounts to a factor of about 0.7. This curve agrees well with the values (shown by black crosses) recalculated from the drop-based experimental data into the equilibrium subsurface concentration. It can be expected that above the threshold value for the surface activity coefficient of *b* = 10 m^3^ mol^−1^ the difference between the drop-based and bubble-based results becomes noticeable.

Figure 1 also illustrates the results obtained for decanol solutions. The surface tension values were measured with the drop profile (filled circles) and bubble profile (open circles) analysis tensiometry in [54]; the data obtained by the bubble profile method almost coincide with the data obtained by the du Noüy ring and Wilhelmy plate methods, as reported in [72]. The bold red curve was obtained by fitting the drop-based experimental values using Eqs. (1), (3), (5) using the parameters summarised in Table 1, while the dependence obtained by the fitting of the bubble-based data using Eqs. (1)–(3) resulted in almost the same model parameters. It should be noted that this dependence (shown by the dash-dotted curve) coincides to within the graphical accuracy with the curve obtained by the recalculation of the drop-based fitting curve on the equilibrium concentration.

**Fig. 2:**
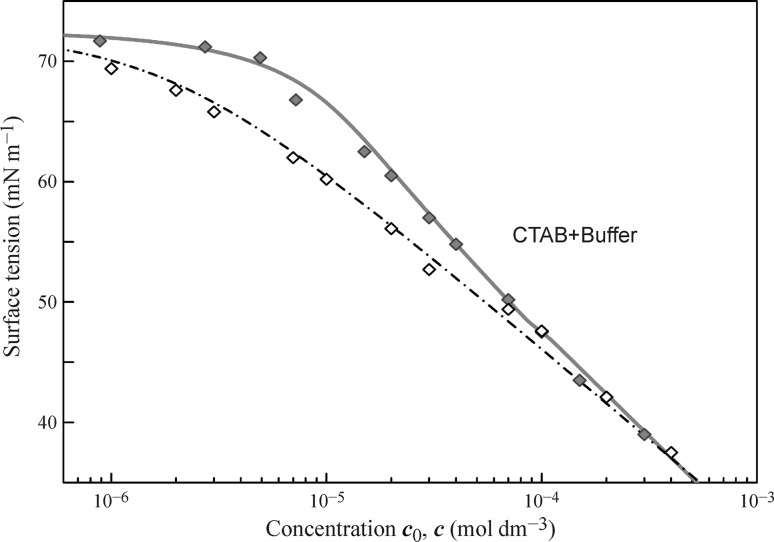
Surface tension isotherms for solutions of CTAB in phosphate buffer with a concentration of 10 mol m^−3^

The experimental values and calculated equilibrium surface tension for CTAB solutions in phosphate buffer (10 mol m^−3^) measured by the two methods, and theoretically calculated, are shown in Fig. 2. The data obtained with the drop profile analysis (filled diamonds) are taken from [75], where a capillary with a radius smaller than that mentioned previously was employed. Therefore, the drop surface area and volume were 14 mm^2^ and 5.4 mm^3^, respectively. These values were used in the calculation with the Frumkin model for an electroneutral surface layer [73]; the *ω*_0_ values listed in Table 1 refer to the total area of each CTAB molecule. In this case, also the dash-dotted curve obtained from fitting the bubble-based experimental data (open diamonds, [54]) coincides with the curve obtained by the recalculation of the drop-based fitting curve on the equilibrium concentration.

**Fig. 3:**
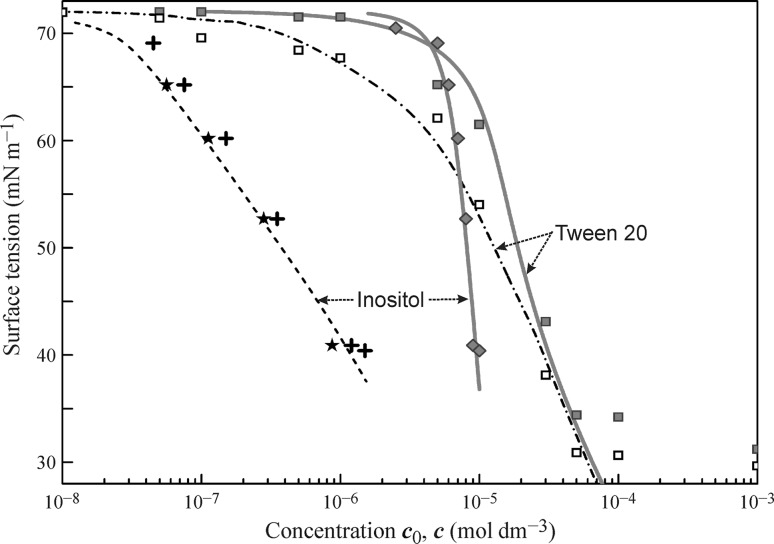
Surface tension isotherms for aqueous Inositol and Tween 20 solutions

Figure 3 illustrates the results obtained in [76] for aqueous solutions of Inositol-C2-PAF. These phospholipids, extensively studied in [77–82], are the main compounds of eukaryotic plasma membranes which undergo rapid and continuous turnover. The resulting lipid metabolites regulate essential signal pathways. They control several cellular processes, such as cell proliferation, apoptosis, metabolism, and migration. The experimental isotherm measured using the drop profile method (filled diamonds) exhibits a very large slope: the concentration increase from 6 to 9 mmol m^−3^ results in a surface tension decrease from 65 to 40 mN m^−1^; the fitting of these data by Eqs. (1)–(3) (bold red curve) results in an unrealistically small *ω*_0_ value. In this case, the depletion of the bulk concentration caused by adsorption should not be neglected, because once a drop for the profile analysis tensiometry is formed, some part of the molecules becomes adsorbed, thus leading to a remarkable depletion of the bulk concentration in the drop. Therefore, correct results from a comparison between experimental data and model calculations can be obtained only if the final concentration of Inositol-C2-PAF inside drop after the establishment of the adsorption equilibrium is calculated from Eq. (4). This leads to a quite realistic value of the area per Inositol-C2-PAF molecule shown in the Table 1.

The adsorption activity of Inositol-C2-PAF is very high: this substance exhibits the largest *b* value among those given in Table 1. Using the adsorption isotherm, a surface tension value of 65 mN m^−1^ corresponds to the equilibrium concentration within the drop *c* 2 orders of magnitude smaller than the initial concentration *c*_0_ of the solution. The asterisks () shown in Fig. 3 correspond to dynamic surface tension after 10^5^ s (when the system equilibration is almost completed) calculated using Eqs. (8)–(11) with the isotherm defined by Eqs. (1), (3), (5) and the model parameters given in Table 1 at initial bulk concentrations of 6, 7, 8, and 9 mmol m^−3^. The equilibrium concentrations *c* in these points differs by factors of 0.0092, 0.0164, 0.0355, and 0.096, respectively, from the corresponding initial concentration values. These values are in a good correspondence with those recalculated from the drop-based isotherm constructed as a function of the equilibrium concentration determined via Eq. (4) (dashed black curve), and with the values (shown by black crosses) obtained by the recalculation of the experimental drop-based data as a function of the equilibrium concentration. Note that the equilibrium concentration values obtained by the two methods (equilibrium and kinetic calculations) almost coincide. This can be ascribed to the fact that the drop radius *R* used in the kinetic calculations with Eqs. (8)–(11) was defined according to the *S*/*V* ratio. In the experiments reported in [76] this ratio was 2.14 mm^−1^; as for a spherical object this ratio is equal to 3/*R*, the radius *R* was taken to be 1.4 mm; for this value the equilibrium concentrations calculated from the drop-based fitting were found equal to the concentrations calculated from the kinetic curve approaching the equilibrium plateau.

Figure 3 illustrates also the experimental and calculated values obtained for Tween 20 solutions [54]; here again the dash-dotted curve referring to the data from bubble profile experiments coincides with the curve obtained by the recalculation of the drop-based fitting curve on the equilibrium concentration.

**Fig. 4:**
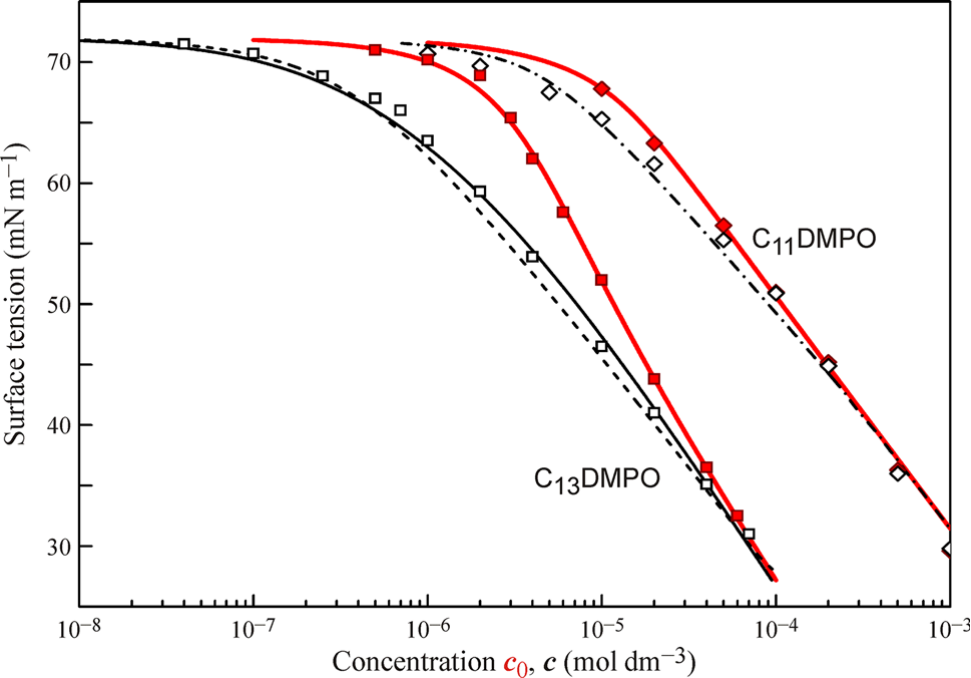
Surface tension isotherms for C_11_DMPO and C_13_DMPO, data from [39, 43, 85, 86, 90]

**Fig. 5:**
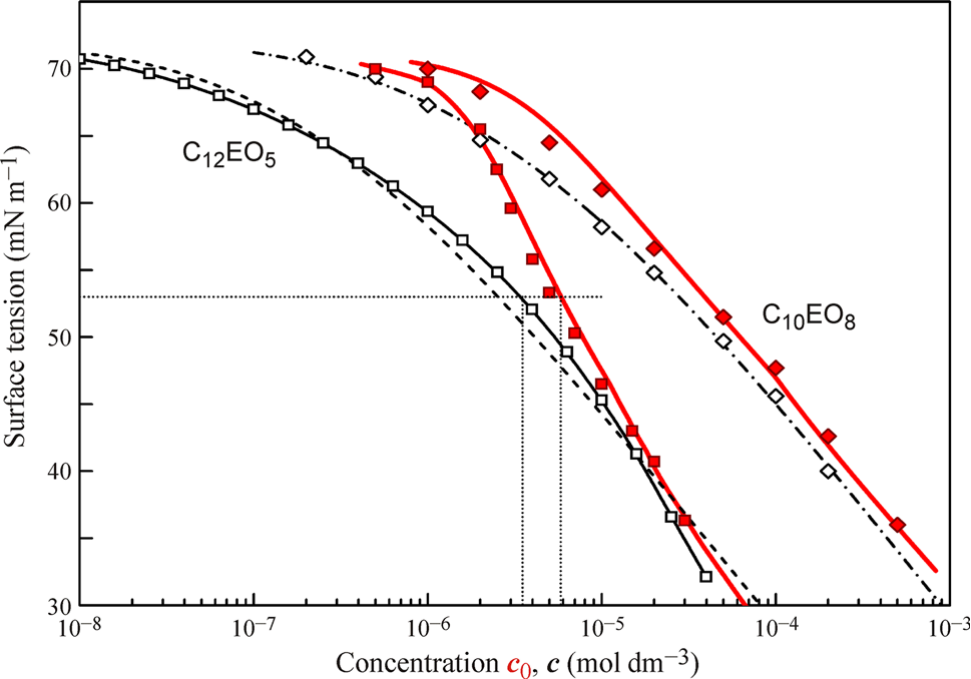
Surface tension isotherms for C_10_EO_8_ and C_12_EO_5_, data from [49, 87–89]

**Fig. 6:**
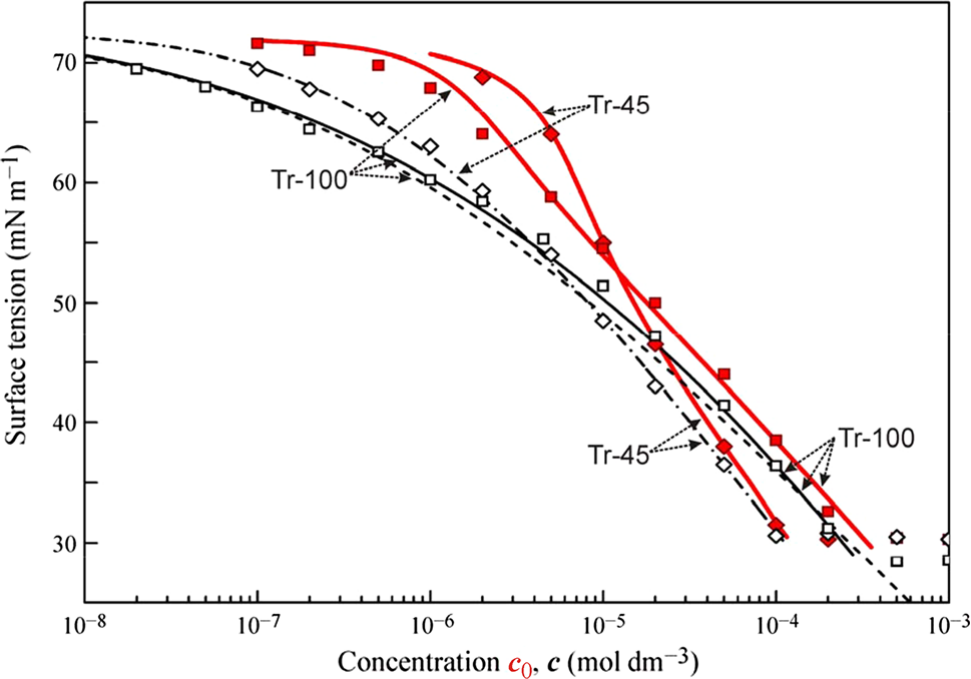
Surface tension isotherms for Triton X45 and X100, data taken from [58, 83]

**Fig. 7:**
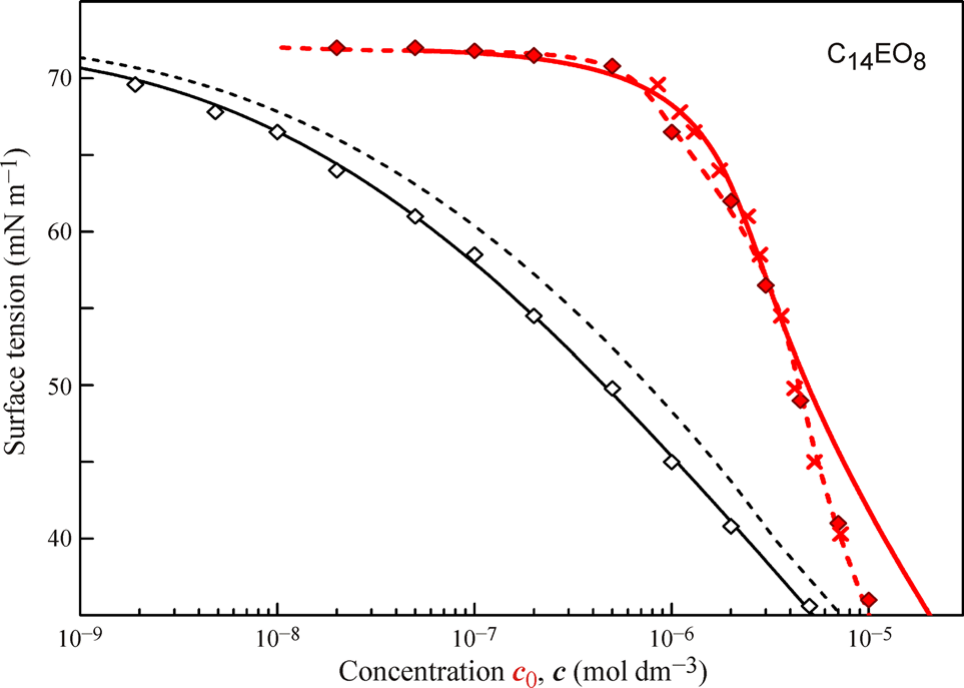
Surface tension isotherms for C_14_EO_8_, data from [40, 48, 65]

In Figs. 4, 5, 6, and 7 the data obtained for the non-ionic surfactants C_11_DMPO, C_13_DMPO, C_10_EO_8_, C_12_EO_5_, Tr-45, Tr-100, and C_14_EO_8_ are summarised, based on the results reported in [39–46, 48, 58, 59, 83–93]. It is seen that for almost all surfactants a good agreement exists between the fitting results of the drop profile and bubble profile analysis data by the Frumkin isotherm using the parameters given in the Table 1.

In Figs. 4, 5, and 6 the drop-based and bubble-based experimental data are shown by filled and open symbols, respectively, and the bold red solid curves illustrate the fitting results of the drop-based data by the Frumkin isotherm with model Eqs. (1)–(3). For the less surface active surfactants C_11_DMPO, C_10_EO_8_ and Tr-45 the black dash-dotted curves correspond to the isotherms obtained by fitting the bubble-based data using the Frumkin isotherm Eqs. (1), (3) (5) with the same parameters given in Table 1. Note, the curves are visually indistinguishable from the corresponding curves obtained by the recalculation of the drop-based fitting curves on the equilibrium concentration. For the more surface active substances C_13_DMPO, C_12_EO_5_ and Tr-100 these two types of curves are somewhat different from each other, as shown by the solid black and dashed black curves, respectively.

The solutions of the highly surface active C_14_EO_8_ (see Fig. 7, data from [40, 48, 65]) are poorly described by the Frumkin model, cf. bold red solid curve); also the dashed black curve, which shows that the isotherm thus fitted and recalculated on the equilibrium concentration does not agree well with the bubble-based experimental data (open diamonds). Thus, the theoretical dependence (black solid curve) obtained by fitting the bubble profile data using the more rigorous reorientation model [44, 45] with parameters *ω*_1_ = 4.4·10^5^ m^2^ mol^−1^, *ω*_2_ = 1.0·10^6^ m^2^ mol^−1^, *α* = 0.9, *b* = 1.0·10^5^ m^3^ mol^−1^, and *ε* = 0.008 m mN^−1^ was calculated, which exhibits much better agreement with the experiment. Also, the red dashed curve obtained by the recalculation of this bubble-based dependence on the initial bulk concentration *c*_0_ is in good agreement with the drop profile analysis experimental data (filled diamonds), which is also true for the values shown by red crosses, obtained by the recalculation of the bubble-based experimental data on the initial bulk concentration *c*_0_.

Note, however, even when the Frumkin adsorption model is not the optimum theoretical basis for describing the experimental surface tension isotherms, the description is at least somehow acceptable. Without considering the depletion effects, however, by just fitting the measured surface tension isotherm using the drop profile tensiometry data, enormous errors can arise, as shown in Table 2 for the three ethoxylated surfactants given in Figs. 5 and 7.

**Table 2:** Model parameters for the Frumkin adsorption isotherm obtained when fitting the drop profile analysis data without taking the depletion effects into account; in parenthesis are the parameter values obtained with consideration of the depletion effects in the single drop

Surfactant	*ω*_0_, 10^5^ m^2^ mol	*b*, m^3^ mol^−1^	*a*	*ε*, 10^−3^ m mN^−1^
C_10_EO_8_	3.0 (4.0)	260 (350)	0.3 (0.2)	3 (9)
C_12_EO_5_	2.0 (3.5)	275 (16,000)	1.2 (−0.9)	3 (5)
C_14_EO_8_	1.8 (3.0)	190 (90,000)	1.8 (−4.7)	3 (6)

As one can easily see, ignoring the depletion effect due to adsorption at the single drop surface leads to tremendous changes, in particular for the parameter *b*, which for C_14_EO_8_ amounts to a factor of 270 and for C_12_EO_5_ of 60. Also, the slope of the isotherm can be much different, leading to different values of *ω*_0_. Again, for C_14_EO_8_ and C_12_EO_5_ the differences are the largest, but even for C_10_EO_8_ the obtained molar area becomes 4 × 10^5^ m^2^ mol^−1^ instead of 3 × 10^5^ m^2^ mol^−1^, which is a 33% larger value.

**Fig. 8:**
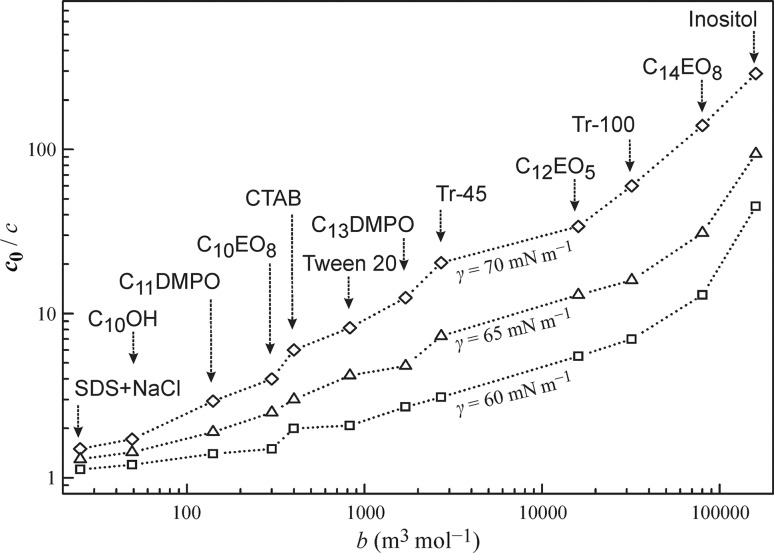
The dependencies of the ratio of initial concentration *c*_0_ to the subsurface equilibrium concentration *c* calculated via fitting the experimental data on the adsorption activity coefficient *b* for three constant surface tension values *γ* as labelled; *dotted lines* are guides for the eye

Figure 8 shows the surfactant concentration depletion in the drop (initial concentration) as compared with the equilibrium concentration, on the surfactant adsorption activity coefficient *b* for three surface tension values. The *b* values are those listed in Table 1, and the concentrations were taken from the results shown in Figs. 1, 2, 3, 4, 5, 6, and 7. The increase of the adsorption activity coefficient corresponds to an increase of the drop to bubble concentrations ratio. Hence, to obtain with a drop the same surface tension as with a bubble it is necessary to have larger initial concentration in the drop-based experiments. This difference becomes smaller with decreasing surface tension, i.e. increasing initial surfactant concentration in the drop.

**Fig. 9:**
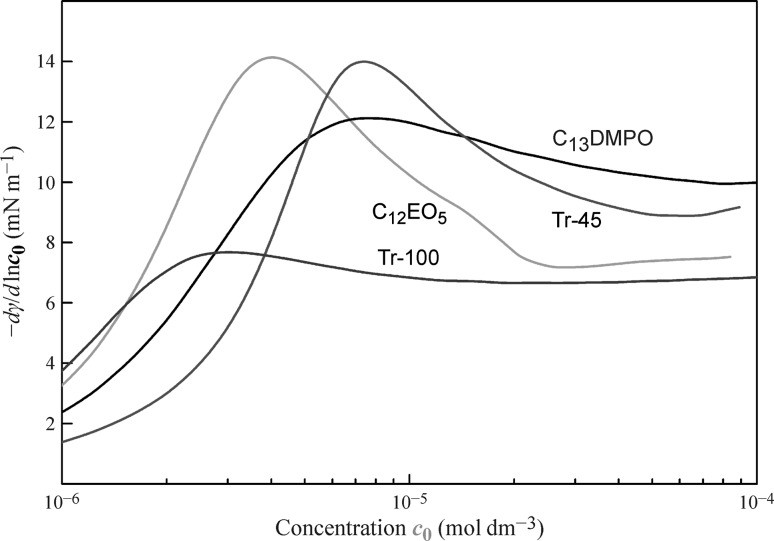
The dependencies of the derivatives ${{ - {\text{d}}\gamma } \mathord{\left/ {\vphantom {{ - {\text{d}}\gamma } {{\text{d}}\ln c_{0} }}} \right. \kern-0pt} {{\text{d}}\ln c_{0} }}$ calculated from the isotherms shown in Figs. 4, 5, and 6, using the drop-based best fit values and plotted against the initial surfactant concentration *c*_0_ for the solutions of C_12_EO_5_, C_13_DMPO, Tr-45, and Tr-100

It is interesting to note that the derivatives ${{ - {\text{d}}\gamma } \mathord{\left/ {\vphantom {{ - {\text{d}}\gamma } {{\text{d}}\ln c_{0} }}} \right. \kern-0pt} {{\text{d}}\ln c_{0} }}$ (i.e. with respect to the initial bulk concentration) for the drop-based isotherms are essentially different from the derivatives ${{ - {\text{d}}\gamma } \mathord{\left/ {\vphantom {{ - {\text{d}}\gamma } {{\text{d}}\ln c}}} \right. \kern-0pt} {{\text{d}}\ln c}}$ (with respect to the equilibrium concentration) which are equal to Γ*RT* and increase monotonously with increasing surfactant concentration. On the contrary, the derivatives $- {{{\text{d}}\gamma } \mathord{\left/ {\vphantom {{{\text{d}}\gamma } {{\text{d}}\ln c_{0} }}} \right. \kern-0pt} {{\text{d}}\ln c_{0} }}$ calculated from the drop-based isotherms for surfactants with the highest surface activity shown in Figs. 4, 5, and 6 exhibits maxima (See Fig. 9). This effect can be explained by the loss of surfactant due to its adsorption at the surface of the drop and shows the importance of using the equilibrium concentrations rather than the initial bulk values.

**Fig. 10:**
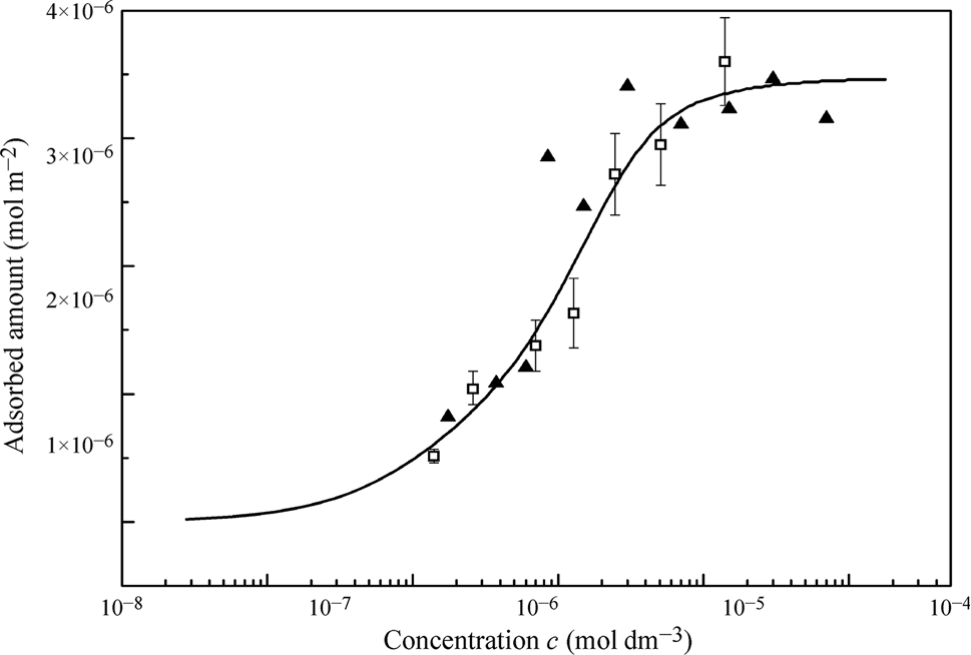
Dependence of the equilibrium adsorption of C_13_DMPO on the equilibrium bulk concentration; *squares* experimental results obtained by the bubble and drop methods; *triangles* values calculated using the Gibbs’ equation; *curve* the values calculated using the Frumkin model with the parameters listed in Table 1

Figure 10 shows as an example, the C_13_DMPO adsorbed amount (squares) plotted vs. the equilibrium bulk concentration; these values were calculated from the mass balance expression Eq. (7) using the drop-based and bubble-based experimental data shown in Fig. 4. In these experiments [43] the ratio *V*_D_/*S*_D_ was 0.6 ± 0.01 mm. The theoretical dependence of adsorption on the equilibrium concentration also presented in Fig. 10 (curve) was derived from the Frumkin adsorption model using the parameters given in Table 1 and is in good agreement with the measured data. This Figure shows also the experimental error related to the adsorption measurements using this method, and the values calculated with the Gibbs’s equation from the experimental isotherm obtained by the du Noüy ring method. It is seen that all results shown in this figure are in a satisfactory mutual agreement. Similar results obtained for C_14_EO_8_ solutions using the reorientation model were reported in [43]. The adsorption values for β-casein and BSA (HSA), obtained using the proposed method, are in a satisfactory agreement with those measured by the ellipsometry and radiotracer techniques [43].

### Dynamic Surface Tensions Measured with Drop Profile Analysis

The diffusion-governed kinetics of adsorption at the drop surface from the solution bulk, and various adsorption mechanisms have been discussed in a number of studies [11–13, 32, 44, 45, 66, 67, 94–100]. The experimental error which involves all possible factors does not exceed ±0.5 mN m^−1^. Using the theory implemented in Eqs. (8)–(11) it is possible to calculate the dynamic adsorption and dynamic surface tension assuming the diffusion-governed adsorption mechanism, with the adsorption model parameters initially derived from the bubble-based experiments, which do not involve the adsorption-related depletion of surfactant molecules from the bulk of the drop.

**Fig. 11:**
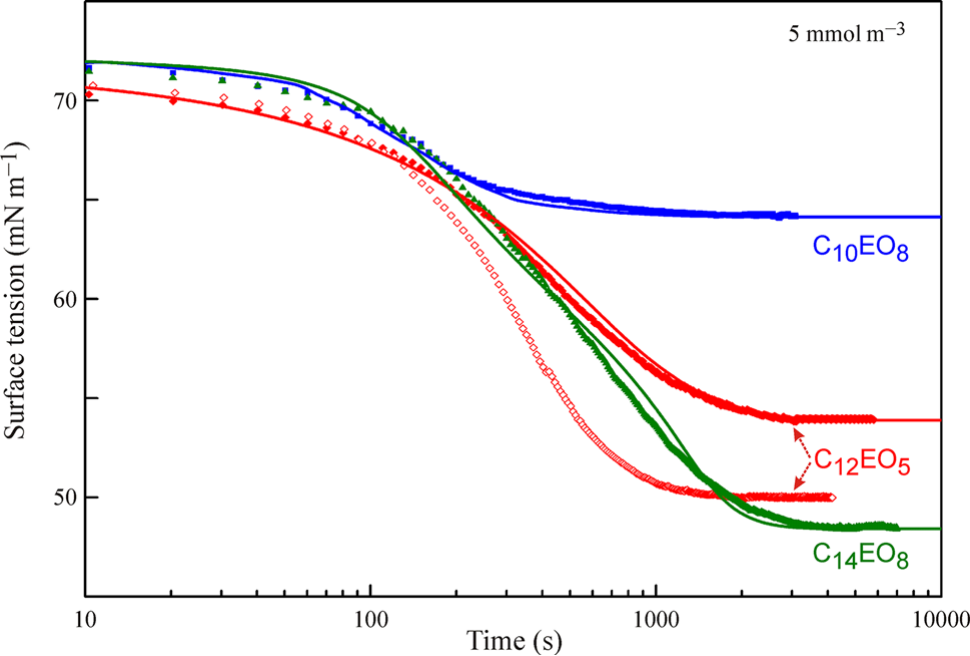
Dynamic surface tension of aqueous solutions of ethoxylated surfactants with an initial concentration of 5 mmol m^−3^; *symbols* experimental data [40, 48, 65, 87–89]; *curves* calculations using Eqs. (8)–(10)

**Fig. 12:**
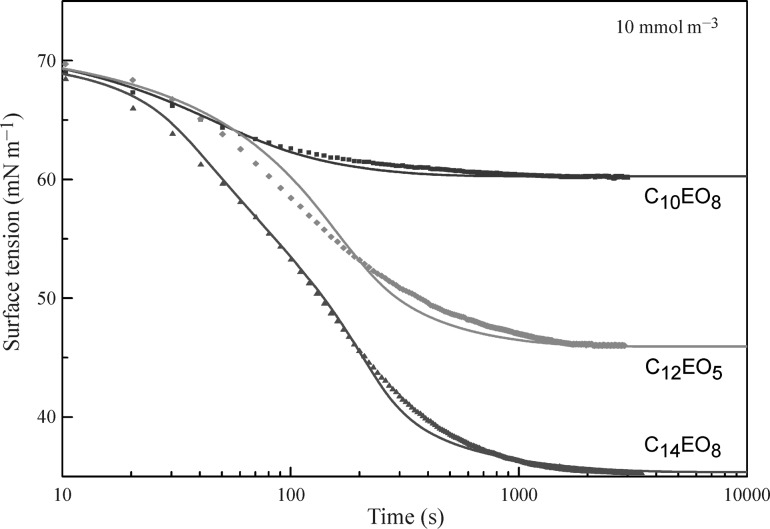
Dynamic surface tension of aqueous solutions of ethoxylated surfactants with an initial concentration of 10 mmol m^−3^; *symbols* experimental data [40, 48, 65, 87–89]; *curves* calculations using Eqs. (8)–(10)

Figures 11 and 12 illustrate the temporal evolution of the surface tension in the drop profile experiments with aqueous solutions of ethoxylated surfactants at two initial concentrations, 5 and 10 mmol m^−3^, respectively, in the drop bulk. It is seen from the experimental data (filled symbols) that at short times (less than about one minute) the surface tension values for different surfactants are quite similar, while at larger times the surfactants which are more surface active exhibit a more pronounced surface tension decrease. Shown in Fig. 11 are also the data obtained for C_12_EO_5_ in bubble profile experiments (open symbols) at the same concentration as the initial concentration in the drop. As expected, the initial surface tension values are quite similar, and with the adsorption progress the drop-related values remain higher than the bubble-related ones because of the gradual depletion of surfactant from the volume of the single drop.

To simulate the theoretical dependencies (solid curves in Figs. 11 and 12) the Fick equation was solved using the boundary condition at the surface given by the reorientation model. For C_14_EO_8_ the parameters values are those given in the discussion of Fig. 7, and for C_12_EO_5_ and C_10_EO_8_ we used the values shown in Table 1. The calculated dynamic surface tension for all solutions show good agreement with the measured data. In the calculations, we used diffusion coefficients in the range (2.0–3.0) × 10^−10^ m^2^ s^−1^ (also shown in Table 1), which were found by best fit. These values are quite realistic, indicating that the adsorption of these non-ionic surfactants obeys the diffusion controlled adsorption mechanism. After the equilibrium is established, the concentration of surfactant in the drop is lower than the initial concentration. The calculations show that for the initial concentrations of 5 and 10 mmol m^−3^ the equilibrium concentration values are exactly the same as those shown in Figs. 5 and 6 as determined using the bubble profile method. In particular, for C_12_EO_5_ at these initial concentrations the equilibrium concentrations were 1.55 and 5.9 mmol m^−3^, correspondingly.

**Fig. 13:**
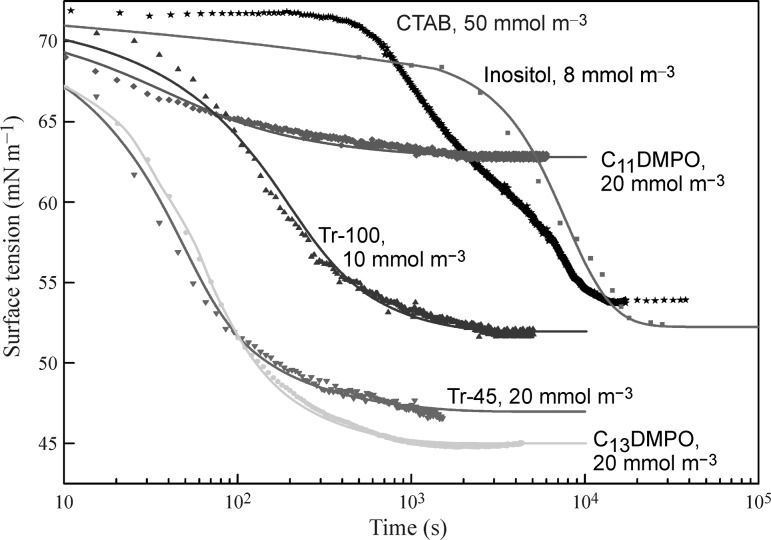
Dynamic surface tension isotherms for aqueous surfactant solutions, experimental data from [39, 43, 58, 76] (*symbols*) and theoretical predictions (*curves*)

Figure 13 illustrates the temporal dependencies of surface tension for several non-ionic surfactants with initial concentrations as labelled; both measured using the drop profile method (symbols) and calculated according to Fick’s equations (curves). Shown are also the experimental results for the cationic CTAB in phosphate buffer (the theoretical curve is not shown because the estimated diffusion coefficient for this substance is by one order of magnitude lower than its realistic value, indicating the presence of a surface barrier or impurities in the solution). It is seen that the decrease in surface tension for CTAB solutions is much slower than that of non-ionic surfactants with lower concentrations; similar behaviour was obtained also for SDS solutions. The theoretical curves calculated with the isotherm parameters and diffusion coefficients listed in Table 1 agree quite well with the experimental findings. The realistic values of the diffusion coefficients indicate that a diffusion-governed adsorption mechanism can be applied for the description of these surfactant systems.

In particular, the dynamic surface tension for the Inositol-C2-PAF solution with a concentration of 8 mmol m^−3^ is well described by the diffusion-governed adsorption model, with a diffusion coefficient estimated via fitting the theoretical prediction to the experimental data to be 4 × 10^−11^ m^2^ s^−1^. This value is realistic for Inositol-C2-PAF molecules having a molecular mass of 716 g mol^−1^.

### Co-Adsorption of Alkanes and Surfactants at Single Drop Surfaces

The co-adsorption of gases or oil vapor from the gas phase at a liquid interface is an important experiment for mimicking the alveoli/air interface in pulmonary systems. In particular, fluorocarbon gases in pulmonary disease therapies were studied. Experimental results and theoretical models for the concurrent adsorption of alkanes from the gas phase and surfactants (or proteins) from their solutions were presented in [49–52, 63, 101–108].

It was shown in [53] that the equilibrium surface tensions of C_10_EO_8_ solution drops with concentrations of 1, 3, and 10 mmol m^−3^ in pure air are 67.5, 63.7, and 59.0 mN m^−1^, respectively, while in air saturated by hexane the corresponding are 50, 47, and 43.4 mN m^−1^, which is 15–17 mN m^−1^ lower. For C_14_EO_8_ solutions the values of the surface tensions for drops with concentrations of 2 and 5 mmol m^−3^ in pure air were 63 and 48.4 mN m^−1^, respectively. At the interface between the solution and air saturated by hexane, however, the surface tensions dropped down by 18–22 mN m^−1^ to become 40.7 and 30.6 mN m^−1^, respectively, for these two solutions. In addition, in [109] the temperature dependence of the adsorption and desorption dynamics of heptane vapor at the drop surface of C_10_EO_8_ solution was studied.

**Fig. 14:**
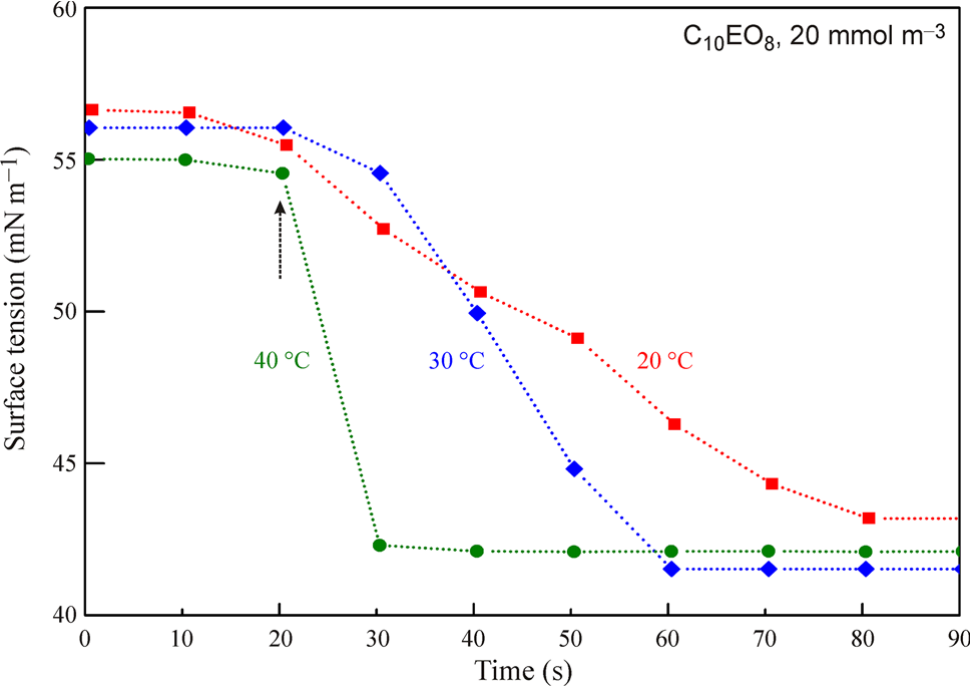
The dynamics of surface tension decrease for a C_10_EO_8_ solution after the injection of heptane into the cell at different temperatures; *dotted lines* are eye guides

It is interesting to compare the rates of surface tension decrease for C_10_EO_8_ solutions after the injection of heptane into the cell at different temperatures. The injection process duration did not exceed 2 s. It is seen from Fig. 14 that the surface decrease rate becomes essentially higher with increasing temperature, approximately by a factor of 5 for the temperature increase from 20 to 40 °C. This can be ascribed not only to the increased heptane vapor concentration in the gas phase (by a factor of 2.5), but also to the decrease of the heptane adsorption activation energy.

### Differences Between the Rheological Characteristics Measured by Bubble vs. Drop Profile Analysis Tensiometry

**Fig. 15:**
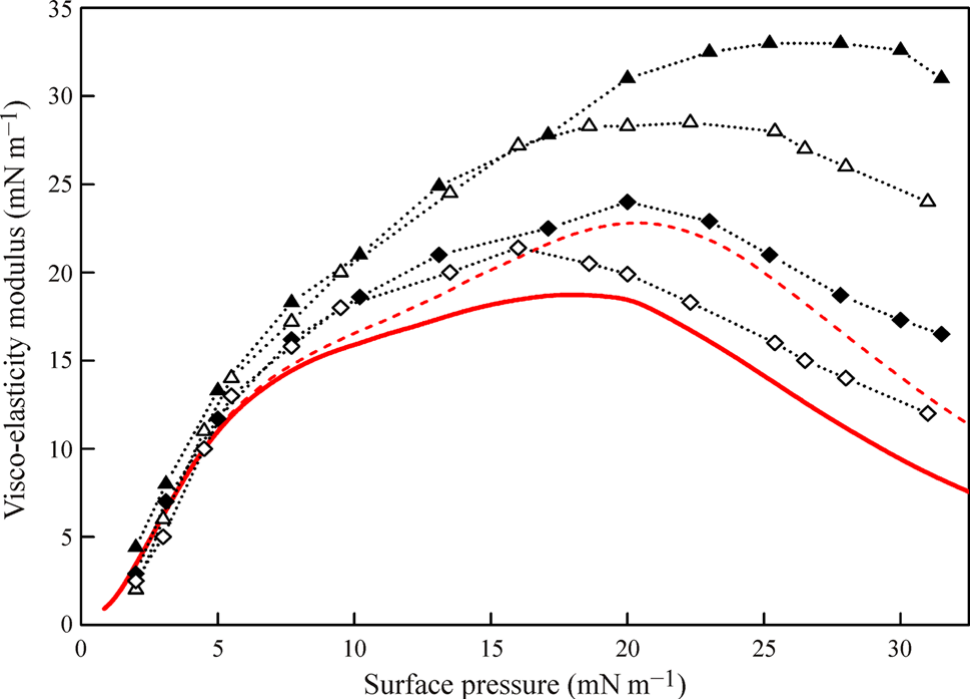
Viscoelasticity modulus as a function of the surface pressure for C_12_EO_5_ solutions at two oscillation frequencies [0.1 Hz (filled triangle, unfilled triangle) and 0.01 Hz (filled diamond, unfilled diamond)], as measured by the drop method (filled triangle, filled diamond) and bubble method (unfilled diamond, unfilled triangle); red curves calculated with the diffusion model for a frequency of 0.01 Hz for the drop method (dashed line) and bubble method (solid line); the data were taken from [46]

The rheological characteristics obtained by the drop profile analysis method for various systems were discussed in [46–48, 110–114]. In [46] the differences in dilation rheology characteristics measured for solutions of various surfactants by bubble and drop profile analysis methods were analysed. The experimental surface tension isotherms for the C_12_EO_5_ solutions measured by the drop profile (filled symbols) and bubble profile (open symbols) analysis tensiometry are shown in Fig. 5. The theoretical curves obtained by fitting of the experimental values with the Frumkin isotherm using the parameters given in Table 1 are shown by solid lines while the dashed lines were calculated for the drop-based data as a function of the equilibrium bulk concentration. From the isotherms, the concentrations could be selected for which the equilibrium surface tension values obtained by the two methods are the same. For thus selected pairs of concentrations, the dilational rheological parameters were measured in [46] in the frequency range between 0.005 and 0.5 Hz. In Fig. 15 the viscoelasticity modulus as a function of the surface pressure Π at two oscillation frequencies (0.1 and 0.01 Hz) is shown as measured by both methods (note, the dotted lines are guides for eyes only). For surface pressure less than 15 mN/m the data are more or less the same, however, for surface pressures larger than 15 mN/m, the viscoelasticity modulus depends on the method used: with the drop profile method, we obtain higher values than with the bubble profile method. Similar findings have been discussed in [115, 116].

Various causes of this phenomenon were analysed in [46]; in particular, the geometric profile (bubble or drop) was considered. On the basis of the diffusion model given by Eqs. (22) and (23) the theoretical dependencies were calculated for the oscillation frequency 0.01 Hz (see Fig. 15). The results for the drop method are given by the red dashed curve, while those for the bubble method by the red solid curve, using a diffusion coefficient of *D* = 4.0 × 10^−10^ m^2^ s^−1^ and *D* = 8.0 × 10^−10^ m^2^ s^−1^ for the drop and bubble experiments, respectively. The reason for the difference in the optimum diffusion coefficients could be caused by convection in the cell for the bubble experiments. A more realistic explanation, however, for the difference in *D* would be again the surfactant mass balance. Upon expansion of the drop surface, additional surfactant molecules adsorb, and therefore, the C_12_EO_5_ bulk concentration decreases, while in contrast upon compression the opposite process happens. This leads to an increase of the dilational modulus.

The mass balance in a single drop at very slow oscillations can be easily given by:

24 $$\Delta c = - \frac{{\Gamma_{e} }}{{\frac{{{\text{d}}\Gamma }}{{{\text{d}}c}} + \frac{{r_{0} }}{3}}} \cdot \frac{\Delta A}{{A_{0} }},$$

where Γ_*e*_ is the adsorption at equilibrium, and *A*_0_ and Δ*A* are the equilibrium surface area and the variation of the surface area. Thus, any variation of the drop surface area entails a finite change in the concentration Δ*c*, which increases when the drop radius *r*_0_ and the derivative dΓ/d*c* decrease. In contrast, for a bubble the concentration variation is negligible.

The surface tension change is related to the concentration change via:

25 $$\Delta \gamma = \frac{{{\text{d}}\gamma }}{{{\text{d}}c}}\Delta c = - \frac{1}{c}\frac{{{\text{d}}\gamma }}{{{\text{d}}\ln c}}\frac{{\Gamma_{e} }}{{\frac{{{\text{d}}\Gamma }}{{{\text{d}}c}} + \frac{{r_{0} }}{3}}} \cdot \frac{\Delta A}{{A_{0} }}.$$

Therefore, we easily see that the surface elasticity of a drop is higher than that of a bubble due to the changes in concentration inside the bulk. With increasing frequencies, these concentration changes become lower and finally negligible.

To investigate this problem, stress deformations with drops and bubbles were performed [46]. It was shown that for low values of the equilibrium surface pressure of C_12_EO_5_ solutions, the stress-related jump of the surface tension as measured by the two methods is almost the same. The experiments at the surface tension equal to 53 mN m^−1^ which correspond to the pair of concentrations 3.5 × 10^−6^ mol dm^−3^ for bubble and 5.6 × 10^−6^ mol dm^−3^ for drop (see dotted lines in Fig. 5 above) have shown that the magnitudes of surface tension jump are 0.9 and 1.3 mN m^−1^, respectively. For these concentrations, the surface tension jump for the drop is higher than that for the bubble for surface oscillation frequencies 0.1 and 0.01 Hz, respectively; with the increase of C_12_EO_5_ concentration the difference between the amplitudes observed in the two experimental methods becomes even more pronounced. Thus, the surface area and volume changes of a drop of a surfactant solution due to sinusoidal perturbations lead to additional changes of the adsorbed amounts and of the bulk concentration. These, in turn, result in an increase of the viscoelasticity modulus. For a bubble formed in a large reservoir of the same surfactant solution, the same changes are negligible so that the viscoelasticity modulus remains unaffected, i.e. its value is lower than that measured with a drop.

Note, however, that this increased viscoelasticity modulus as measured by drop profile tensiometry, is only obtained for surfactants of sufficiently high surface activity. The viscoelasticity modulus for a large number of surfactants, such as C_10_EO_5_, C_10_EO_8_, C_14_EO_8_, C_13_DMPO, SDS and the Tritons X-45, X-100, X-165, and X-405, have been discussed [47], and it was shown that the differences in the values obtained by the drop and bubble profile analysis methods, respectively, can be quantitatively explained by the presented theory.

## Conclusions

In this review, we analysed studies of surface tension isotherms and dilation viscoealsticity of surfactants using the bubble and drop profile analysis tensiometry. The analysis includes also systems which deal with the co-adsorption of the surfactant molecules from the solution drop bulk and alkane molecules from the air phase around saturated by alkane vapor. The experimental data from literature for ionic (SDS and CTAB) and nonionic (C_10_OH, C_11_DMPO, C_13_DMPO, Tr-45, Tr-100, Tween 20, Inositol, C_10_EO_8_, C_12_EO_5_ and C_14_EO_8_) surfactants are analysed. We propose here a protocol for correcting the adsorption and surface tension data obtained from drop profile analysis experiments, which is based on the depletion of surfactant molecules from the bulk of the drop due to adsorption at the drop surface. The procedure is validated for aqueous solutions of various surfactants having different surface activities and can be described by the generalized Frumkin adsorption model. Using other adsorption models, the same protocol would lead to similar results.

The dynamic surface tensions of the mentioned non-ionic surfactants, measured using the drop profile analysis method, were compared with values calculated via Fick’s diffusion equation. A good agreement was obtained with quite realistic values for the diffusion coefficient of (2.0–3.0) × 10^−10^ m^2^ s^−1^, indicating that the adsorption of these surfactants obeys the diffusion mechanism. Also, the surfactant concentration within the drop after the equilibration calculated via the dynamic modelling process was found to be lower than the initial one, and equal to that calculated with the surfactant solution depletion due to the adsorption taken into account.

The influence of the adsorption of alkanes at the drop surface from the ambient air phase saturated by alkane vapor on the surface tension of water or aqueous surfactant solution is discussed. In particular, it is found that the surface tension of C_10_EO_8_ solution drops with concentrations of 1–10 mmol m^−3^ at the interface with pure air is in the range between 67.5 and 59.0 mN m^−1^, while at the interface with air saturated by hexane the surface tension values are lowered by 15–17 mN m^−1^. For C_14_EO_8_ solutions at the interface with hexane saturated air the surface tension becomes even lowered by 18–22 mN m^−1^. A theoretical model was proposed in [65] to describe such systems assuming a multilayer adsorption of alkane and a diffusion mechanism controlling both the adsorption of surfactant from the drop bulk and the adsorption of alkane from the gas phase. This assumption, however, results in unrealistically low diffusion coefficients for alkanes; therefore, a modified model was discussed which involves a kinetic mechanism for the double layer adsorption of alkane which leads to quite realistic values of the alkane adsorption rate constant.

The drop and bubble profile analysis tensiometry was applied also to measure the dilational viscoelasticity of surfactant adsorption layers. Again, significant differences in particular for surfactants of high surface activity were observed. For the adsorption layers of the non-ionic surfactant C_12_EO_5_, for example, the dilational viscoelasticity moduli measured by the two profile tensiometry methods had similar values only when the equilibrium surface pressure was sufficiently small, i.e. Π < 15 mN/m. When the surface pressure values were higher than this value, the viscoelasticities obtained from drop measurements were significantly larger than those obtained from bubble experiments.
